# Peroxiredoxins and Immune Infiltrations in Colon Adenocarcinoma: Their Negative Correlations and Clinical Significances, an In Silico Analysis

**DOI:** 10.7150/jca.38057

**Published:** 2020-03-04

**Authors:** Xiuzhi Zhang, Fenglan Gao, Ningning Li, Jinzhong Zhang, Liping Dai, Hongmei Yang

**Affiliations:** 1 Department of Pathology, Henan Medical College, Zhengzhou, Henan Province, China; 2 Medical Laboratory Center, Henan Medical College, Zhengzhou, Henan Province, China; 3 Henan Institute of Medical and Pharmaceutical Sciences, Zhengzhou University, Zhengzhou, Henan Province, China

**Keywords:** peroxiredoxins (PRDXs), immune infiltration, colon adenocarcinoma (COAD), diagnosis, prognosis

## Abstract

**Background**: Peroxiredoxins (PRDXs) were reported to be associated with inflammation response in previous studies. In colon adenocarcinoma (COAD), however, their correlations and clinical significance were unclear.

**Methods**: The RNA-seq data of 452 COAD patients with clinical information was downloaded from The Cancer Genome Atlas (TCGA) and transcripts per million (TPM) normalized. Comparisons of relative expressions of PRDXs between COAD tumor and normal controls were applied. PRDXs dy-regulations in COAD were validated via Oncomine, Human Protein Atlas (HPA) and Gene Expression Omnibus (GEO) repository. Through Tumor Immune Estimation Resource (TIMER), the immune estimation of TCGA-COAD patients was downloaded and the dy-regulated PRDXs were analyzed for their correlations with immune infiltrations in COAD. The TCGA-COAD patients were divided into younger group (age≤65 years) and older group (age>65 years) to investigate the prognostic roles of age, TNM stage, dy-regulated PRDXs and the immune infiltrations in different age groups through Kaplan-Meier survival and Cox regression analyses.

**Results**: Three of the PRDX members showed their expressional differences both at protein and mRNA level. PRDX2 was consistently up-regulated while PRDX6 down-regulated in COAD. PRDX1 was overexpressed (mRNA) while nuclear absent (protein) in the tumor tissues. PRDX1 overexpression and PRDX6 under-expression were also shown in the stem-like colonospheres from colon cancer cells. Via TIMER, PRDX1, PRDX2, and PRDX6 were found to be negatively correlated with the immune infiltrations in COAD. Both in the younger and older patients, TNM stage had prognostic effects on their overall survival (OS) and recurrence-free survival (RFS). CD4^+^ T cell had independent unfavorable effects on OS of the younger patients while age had similar effects on RFS of the older ones. CD8^+^ T cell was independently prognostic for RFS in the two groups.

**Conclusions**: Late diagnosis indicated poor prognosis in COAD and dy-regulated PRDXs w might be new markers for its early diagnosis. Age was prognostic and should be considered in the treatments of the older patients. Dy-regulated PRDXs were negatively correlated with immune infiltration levels. CD4^+^ T cell and CD8^+^ T cell infiltrations were prognostic in COAD and their potential as immune targets needed further investigation.

## Introduction

Colorectal cancer was one of the most common cancers in the world and its prognosis was very poor when diagnosed at a late stage. In the United States, as one of the top-3 malignancies, the 5-year relative survival rate of the colorectal cancer patients with localized tumors was 90.3% while dropped to 70.4% and 12.5% when the tumors spread to adjacent and distant organs, respectively 1. In China, as one of the top-10 malignancies, there were 376.3 thousands new colorectal cancer cases and 191.1 thousands patients died of the disease due to late stage and lack of effective treatments 2. Considering the current status of diagnosis and prognosis of colorectal cancer, it is important to find effective early diagnostic markers and therapeutic targets.

Peroxiredoxins (PRDXs), a family of peroxidases, is composed of six PRDX isoforms (PRDX1 to PRDX6) which played important roles in anti-oxidation processes 3, 4. According to the number of cysteines they contain, the PRDXs fall into two categories. PRDX6, which contains one cysteine residue, is called 1-Cys PRDX while the other five members all contain two and they are 2-Cys PRDXs. Unlike the 2-Cys PRDXs, PRDX6, the bifunctional enzyme with peroxidase activity and Ca2+-independent phospholipase A2 (iPLA2) activity, does not use thioredoxin as the electron donor in its catalytic cycle 5-8. The antioxidant properties of PRDXs have been widely studied in cells and animal models. In 1991, higher oxidative stress was first reported in several human tumor cells 9. Subsequently, two oncogenes, Bcr-Abl 10 and Ras 11 were found to be able to increase the hydrogen peroxide generation and the deletion of tumor suppressor gene P53 12 could also increase the oxidative stress. Along with the awareness of the complex relationship between oxidative stress and tumorigenesis, more and more researchers focused their studies on the PRDXs to find their roles during tumor progression 13.

In fact, abnormal expression of PRDXs was reported in many tumors in recent years. In breast cancer, over-expression of PRDX1 14, 15, PRDX2 16, PRDX3 17, and PRDX6 18 was shown. In lung cancer, elevated PRDX1 and PRDX4 were demonstrated to be associated with tumor progression 19, 20. For PRDX2, its important role in the survival of lung cancer cells was also observed 21. As for PRDX6, its activity was indicated to be crucial for lung tumorigenesis 22. In esophageal cancer, PRDX1 was reported to be up-regulated and was considered as tumor-associated antigen 23. While, for PRDX6, it was down-regulated in esophageal cancer tissues compared with the normal esophageal tissues. PRDXs was also shown to be dy-regulated or associated with tumor progression in liver cancer 24-26, gastric cancer 27, 28, prostate cancer 29, cervical cancer 30, and oligodendroglial tumors 31. When it came to colorectal cancer, although dy-regulation of PRDXs was shown in many studies 32-35, they were studied separately and their clinical significances were not analyzed systemically. The unique study 36 in colorectal cancer which focused on dy-regulation of PRDXs only included 32 tumor samples and the small sample size limited its reliability. Furthermore, in most of the studies above, colon cancer and rectum cancer were mixed, considering the heterogeneity in colorectal cancer 37, 38, it is necessary to study them separately.

Considering the multiple roles of PRDXs in inflammation 39-41 and the associations between inflammation and cancer 42, 43, there might be significant correlations between PRDXs and immune infiltrations in many tumors. Although the prognostic values of immune infiltration in colorectal cancer has been reported in one study 44, only the percentage of immune cells was analyzed and the correlations between PRDXs and infiltrations were not investigated. Since there might be significant differences among the immune infiltration levels of different patients, the corresponding conclusions deduced from the percentage of the immune cells might be not as reliable as those from the immune cell levels themselves 45. In this study, we focused on PRDXs and the infiltrations of six immune cells (B cell, CD4+ T cell, CD8+ T cell, macrophage, neutrphil, and dendritic cell) in COAD to investigate their correlations and clinical significance systemically. In contrast to the previous studies 32-36, all the PRDXs members were investigated and their dy-regulations were confirmed both at mRNA and protein level; the dy-regulated PRDXs were analyzed for their potential roles and their correlations with the immune infiltrations in COAD. Furthermore, the prognostic effects of the dy-regulated PRDXs and the immune infiltrations were evaluated. Considering the differences of the overall immune response among different COAD patients, the abundance of the six kinds of immune cells which were used for survival analyses would be more reliable than their percentages. We expected that the dy-regulated PRDXs and the immune cells with clinical significance might be new markers or therapeutic targets for COAD.

## Materials and Methods

### Data collection and processing from TCGA

The gene expression data (transcriptome profiling, counts) of COAD patients (n=456) were downloaded from the cancer genome atlas (TCGA) (https://portal.gdc.cancer.gov/). The immune infiltration estimates including the infiltrations of B cell, CD4^+^ T cell, CD8^+^ T cell, macrophage, neutrphil, and dendritic cell of the TCGA-COAD dataset were downloaded from TIMER (Tumor Immune Estimation Resource) 45. Since the clinical information was not available for two patients and there were no following-up data for another two, only 452 of the 456 patients in TCGA-COAD dataset (with 452 primary tumor samples and 41 corresponding normal control samples) were used for analyses in this study while the patients with no clinical information or following-up data were excluded. The clinical information of the 452 patients was shown in Table 1. For normalization, the counts data of all genes in each sample were transformed to transcripts per million (TPM) values 46 for all the samples and the values of PRDXs were extracted for further analyses.

### Expression comparisons of PRDXs in COAD datasets

To ensure the reliability of the results, paired tumor (n=41) and normal (n=41) samples from the same patients in TCGA-COAD dataset were used for expressional comparisons of PRDXs (mRNA) with paired samples T tests. The expressional differences of PRDXs between all the COAD tumors and normal controls in TCGA-COAD dataset were also analyzed through TIMER to validate the efficiency of the paired samples T tests. One-way ANOVA analysis was used to investigate the expressional differences of the PRDXs in COAD patients among different stages. SPSS 18.0 was used and *p*<0.05 was considered to be significant.

The expressional differences of the PRDXs in mRNA level were validated in other COAD datasets through Oncomine database^47^. The filters were set as follows: analysis type: colon adenocarcinama vs. normal analysis; data type: mRNA; *p*-value: 0.05. At protein level, the dy-regulated PRDXs were also investigated for their expressions and locations in COAD and normal colon tissues via Human Protein Atlas (HPA) (https://www.proteinatlas.org/). Chi- square test (method: Likelihood Ratio) was used for their expressional comparisons and *p*<0.05 was considered to be significant.

### Expressions of the dy-regulated PRDXs in colonospheres from primary colon cancer cells

It was reported that compared with parental cells, colonospheres from primary COAD cell line HT29 expressed higher expression of various stemness genes while lower expression of differentiation markers and were more capable of tumor formation 48. Here, to investigate the potential roles of the dy-regulated PRDXs in driving COAD tumorigenesis, the confirmed genes above were analyzed for their expressional differences between HT29 cell line (parental, control) and its colonospheres (HT29, 1st spheres) in GDS4511 from GEO database. The GEO2R (http://www.ncbi.nlm.nih.gov/geo/geo2r) was used for the comparisons. Benjamini and Hochberg (the false discovery rate) was applied to adjust the p values and adjusted p value (adj.*p*)<0.05 was considered significant.

### Function enrichments of the correlated genes with dy-regulated PRDXs

In many studies, co-expression analysis was confirmed to be useful in uncovering new functions or potential roles for specific genes or proteins 49-54. Based on this, we speculated that the genes correlated with the PRDXs might play similar (positively correlated) or opposite (negatively correlated) roles in the same biological processes. Here, the genes correlated with dy-regulated PRDXs in TCGA-COAD dataset were downloaded from cBioPortal (http://www.cbioportal.org/) and the top 20 genes (based on the spearman correlation coefficient) positively and negatively correlated with each PRDX were used for function enrichment analysis through online tool Metascape^55^ individually, to explore the potential roles of the PRDXs during COAD development.

### Correlation analysis between dy-regulated PRDXs and immune infiltrations

Through TIMER, the purity-adjusted Spearman correlations between dy-regulated PRDXs and the six kinds of immune cell infiltrations were analyzed. Through its “SCNA” module, the comparisons of tumor infiltration levels among COAD tumors with different somatic copy number alterations (SCNAs) of the dy-regulated PRDXs were also investigated.

### Survival analyses of the COAD patients

Kaplan-Meier analysis was used to investigate the prognostic roles of the clinical characters (gender, age at diagnosis, histological type, and TNM stage) of the patients both in overall survival (OS) and recurrence-free survival (RFS). Considering the prognostic effects of age (at diagnosis), the 452 patients were divided into younger group (age ≤ 65 years) and older group (age > 65 years) for further analysis. The prognostic roles of the dy-regulated PRDXs and immune infiltrations in each age group were evaluated through Kaplan-Meier analysis. According to the median expression of each PRDX (or the median infiltration abundance of the immune cells), the patients in each age group were divided into high expression and low expression subgroup (for immune cells, high infiltration and low infiltration subgroup). TNM stage was also evaluated for its effects on OS and RFS. To find independent prognostic factors for COAD patients and construct Cox regression models for OS and RFS in different age groups, multi-variable Cox regression analysis (Forward, Likelihood Ratio) was applied with age and the variables (the dy-regulated PRDXs, immune infiltrations, and TNM stage) with *p*<0.05 in Kaplan-Meier analysis. For multi-variable Cox regression analysis, only TNM stage was used as categorical covariate, other variables were continuous. According to the Cox regression models 56, the risk scores (for OS and RFS) of the COAD patients were evaluated. With the median risk scores in each age group, the patients were divided into low risk subgroup and high risk subgroup and their differences in OS and RFS were investigated through Kaplan-Meier survival analyses to validate the efficiency of the Cox regression models. SPSS 18.0 was used and *p*< 0.05 was considered significant.

## Results

### Expressional differences of PRDXs

**Figure 1:**
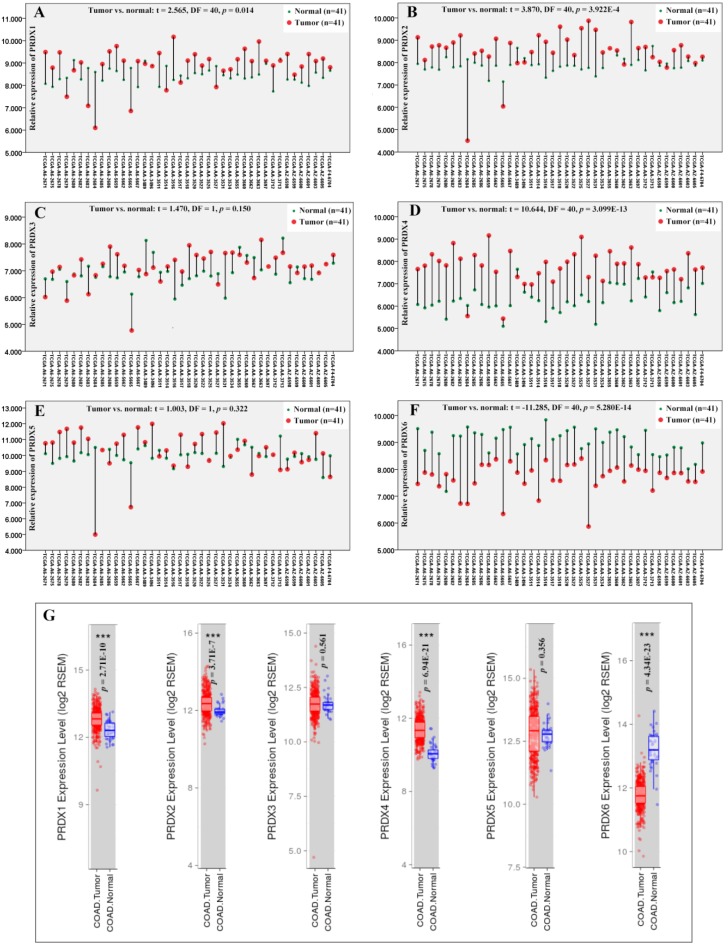
Expressional comparisons of PRDXs between COAD tumors and normal colon tissues. (A)-(F), comparisons of PRDX1, PRDX2, PRDX3, PRDX4, PRDX5, and PRDX6 in paired COAD tumor and normal colon tissues form the same patients in TCGA-COAD dataset through paired samples T test. Y-axis represented the relative expression of the genes and the x-axis represented the 41 COAD patients with paired tumor and colon samples. (G), the expressional comparisons of the PRDX members between all the tumor and normal control tissues in TCGA-COAD dataset through TIMER. For all the analyses, *p* < 0.05 was considered to be statistically significant.

Through paired samples T tests (Figure 1), PRDX1 (*p*=0.014), PRDX2 (*p*=3.922E-4), and PRDX4 (*p*=3.099E-13) were shown to be up-regulated while PRDX6 (*p*=5.208E-14) down-regulated in 41 COAD tumors than their paired normal controls. However, no expressional significance of PRDX3 (*p*=0.150) and PRDX5 (*p*=0.322) was shown, consistent with their expressional differences between all the tumor samples and the normal controls in TCGA-COAD dataset via TIMER (Figure 1G), indicating the reliability of the paired samples T tests. When one ANOVA was applied, however, no significant expressional differences of them (PRDX1, PRDX2, PRDX4, and PRDX6) was shown in COAD of different stages (*p* value for the four PRDXs was 0.895, 0.423, 0.362, and 0.102, respectively).

Through Oncomine database, after applying the filters, other four COAD datasets including Notterman colon 57, Alon colon 58, Ki colon 59, and Kaiser colon 60 were selected for validation of expressional differences of the four PRDXs above. As shown in Table 2, up-regulation of PRDX1 and down-regulation of PRDX6 in COAD were confirmed in all the four datasets. Since no expressional data of PRDX4 was available in Alon colon, it was only confirmed to be increased (in COAD tumors) in the other three datasets. For PRDX2, its up-regulation was shown in three COAD datasets while no significance was seen in Ki colon (*p*=0.154), indicating some heterogeneity of its expression. Generally, no opposite results were shown for all the four PRDXs and the consistency of the expression profiles of the PRDXs was obvious in most of the datasets.

**Figure 2:**
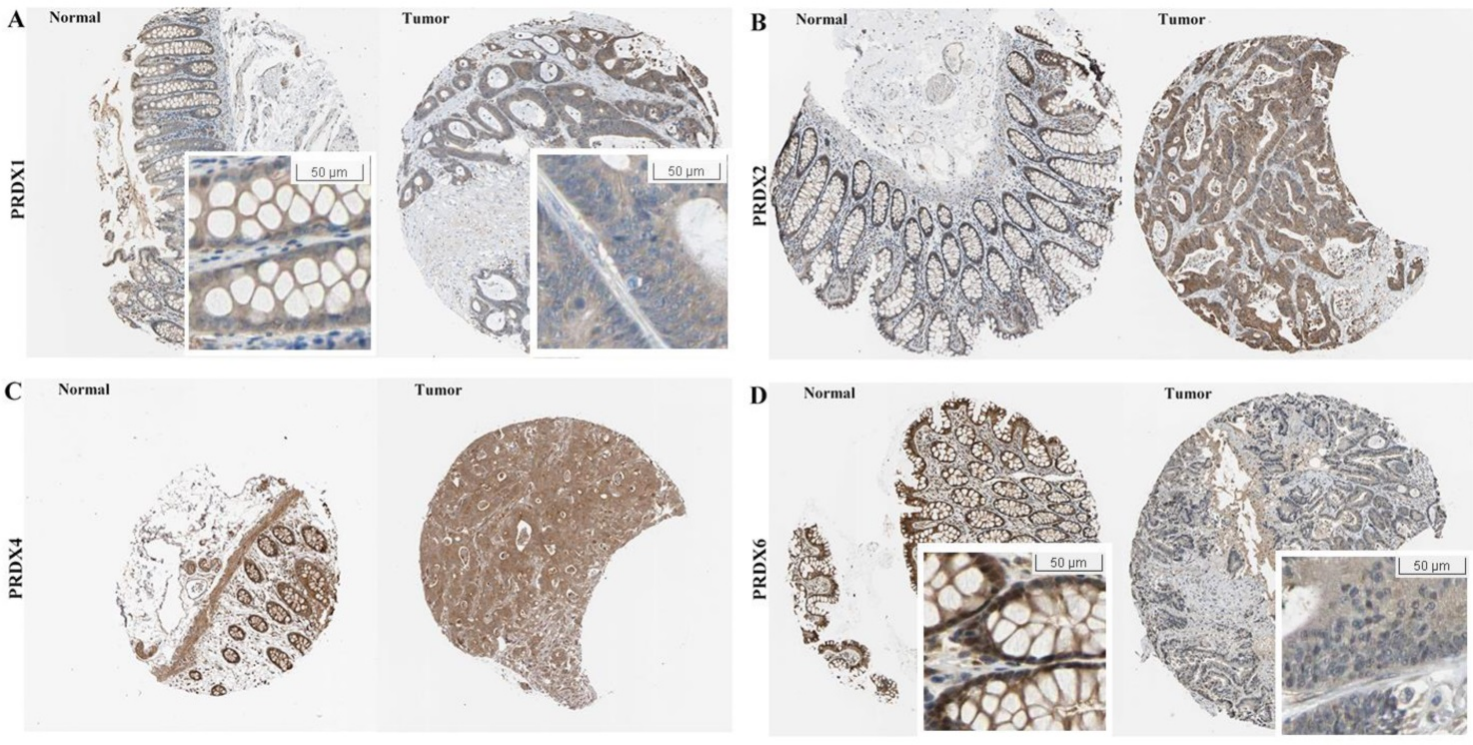
Expressional comparisons of PRDX1, PRDX2, PRDX4, and PRDX6 between normal colon and COAD tissues in HPA database. (A) PRDX1 was positively expressed in cytoplasm/membrane and nucleus of normal glandular cells while prominently in cytoplasm/membrane of COAD tumor cells, only cytoplasm/membrane; (B) Higher expression of PRDX2 in COAD tumors (strong expression) than normal colon tissues (moderate expression); (C) PRDX4 was strongly expressed both in normal colon and COAD tissues; (D) Lower expression of PRDX6 in the COAD tumors (weak expression, with nuclear absence) than the normal colon tissues (strong expression).

At protein level, the immunohistochemical (IHC) data of the four PRDXs above were analyzed via HPA database. For PRDX1 (Figure 2A), it was shown to be moderately expressed in glandular cells of normal colon tissues with cytoplasmic/membranous and nuclear locations. However, in COAD, although moderate expression of PRDX1 was also shown in the tumor cells, the location was different and PRDX1 was found to be positive only in the cytoplasm/membrane of the tumor cells in all the eight COAD tissues. For PRDX2 (Figure 2B), its moderate expression in glandular cells was shown in all the three colon tissues while in tumor cells, its strong expression was shown in six of the seven COAD tissues (Likelihood Ratio = 7.719, *p*=0.005), consistent with it up-regulation in COAD in mRNA level in above analyses. For PRDX4 (Figure 2C), it was shown to be strongly expressed in the glandular cells of the three normal colon tissues and this strong expression was also shown in the tumor cells of seven of the eight COAD tissues, with no significant difference (Likelihood Ratio = 0.674, *p* = 0.412). When it came to PRDX6 (Figure 2D), it was shown to be strongly expressed in the glandular cells of the normal colon tissues while seven of the eight COAD tissues showed its moderate or weak expressions (Likelihood Ratio = 6.189, *p* = 0.045), consistent with its down-regulation in mRNA level. In addition, comparing with the cytoplasmic/ membranous and nuclear location of PRDX6 in normal cells, three of the COAD tissues showed an absence of nuclear PRDX6 in their tumor cells.

From the analyses above, among the six PRDXs, only PRDX1, PRDX2 and PRDX6 were shown to be dy-regulated both at their mRNA and protein level. The three were focused in the following analyses.

### Expressions of the dy-regulated PRDXs in colonospheres from primary colon cancer cells

As shown in Table 3, higher expression of PRDX1 while lower expression of PRDX6 was shown in the colonospheres than their parental cancer cells (HT29), consistent with their expressional differences between normal colon tissues and COAD tumors, indicating their potential functions in driving COAD tumorigenesis. However, no significant expressional difference of PRDX2 was shown between the colonospheres and their parental HT29 cancer cells.

### Functional enrichments of the genes correlated with dy-regulated PRDXs

**Figure 3:**
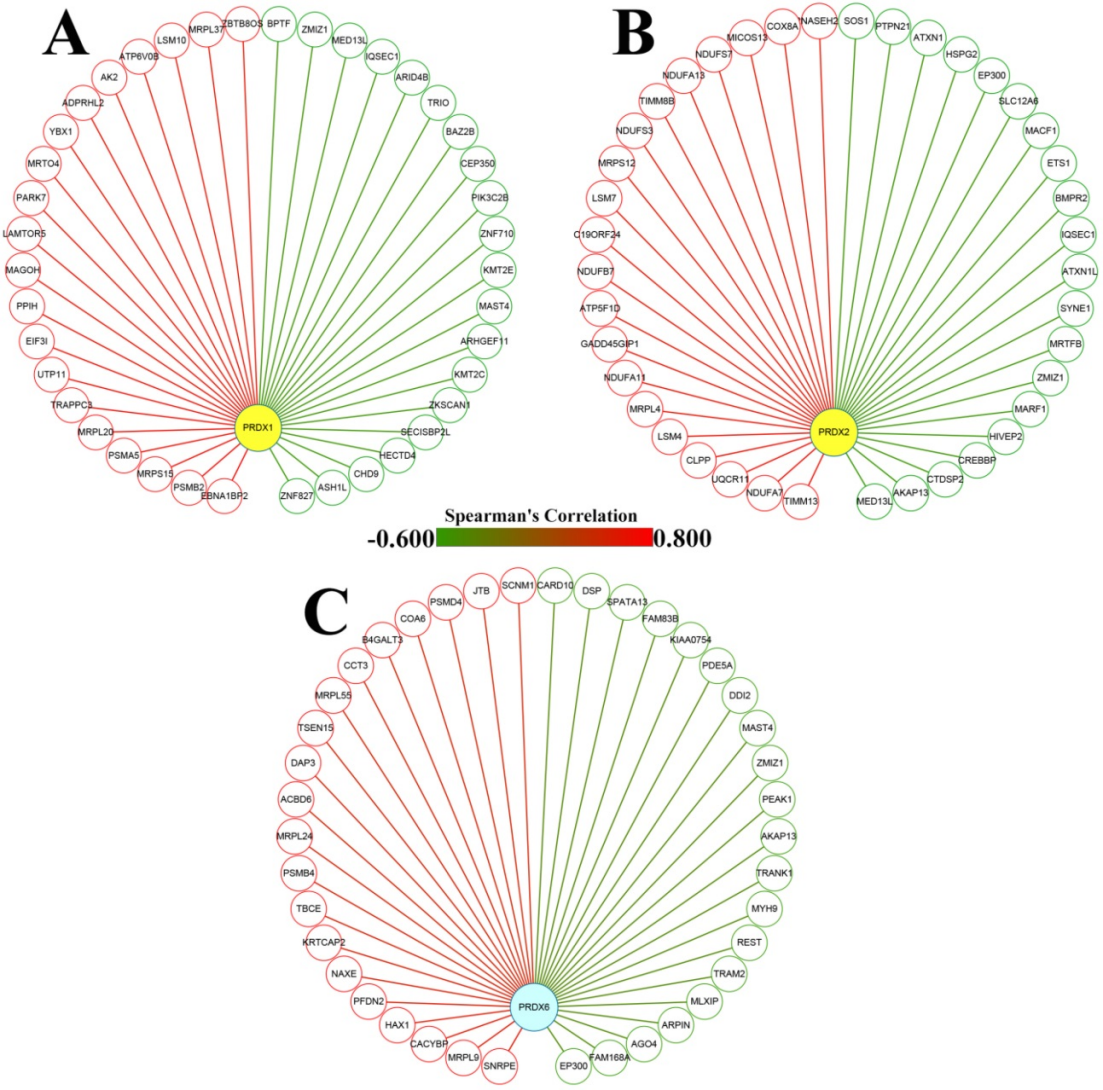
Dy-regulated PRDXs and their correlated genes in COAD. (A)-(C) The nodes represented the top 20 genes positively (red nodes) and negatively (green nodes) correlated with PRDX1, PRDX2, and PRDX6, respectively; the edges indicated the Spearman's correlations between the genes (from -0.600 to 0.800).

**Figure 4:**
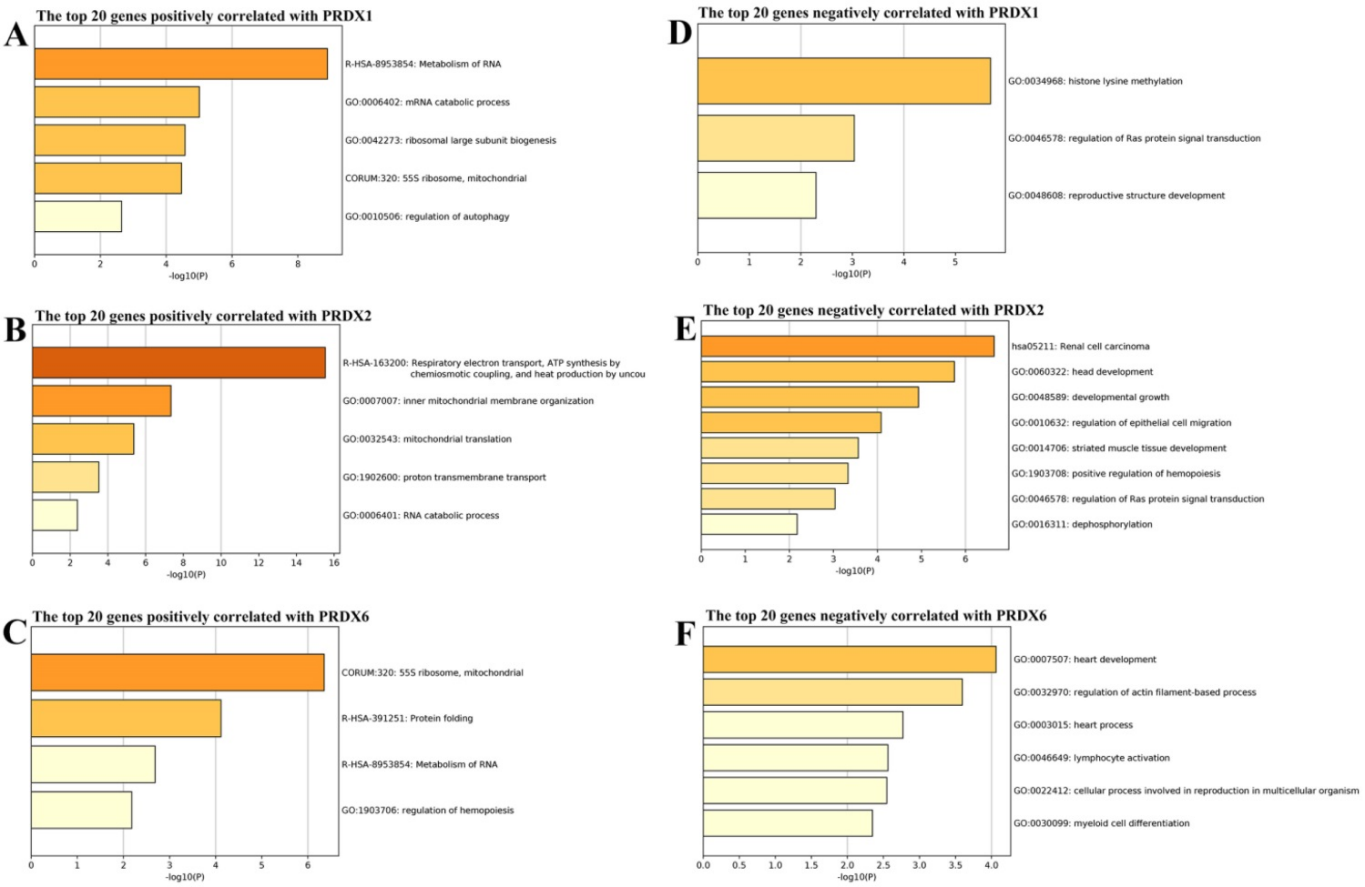
Function enrichments of the correlated genes with dy-regulated PRDXs. (A)-(C) represented the major terms enriched by the top 20 positively correlated genes of PRDX1, PRDX2, and PRDX6, respectively. (D)-(F) indicated the major terms enriched by the top negatively correlated genes with PRDX1, PRDX2, and PRDX6, respectively. Only the terms with *p* < 0.01 were shown in the graphs.

From cBioPortal database, the genes correlated with PRDX1, PRDX2, and PRDX6 in TCGA-COAD dataset were downloaded and the top 20 genes positively and negatively correlated with them were extracted separately (Figure 3) and then applied to functional enrichment analysis via Metascape individually. As shown in Figure 4, the major terms enriched by genes correlated with the three PRDXs were shown. There were some terms in common: RNA metabolism and mitochondrial related processes (enriched by the positively correlated genes of them); Ras protein signal transduction (enriched by the negatively correlated genes of PRDX1 and PRDX2). We speculated that the PRDXs might be associated with these processes. Differences also existed among their enriched terms, indicating their potential roles in some specific terms. For example, PRDX1 might have some function in regulation of autophagy while PRDX2 might be associated with regulation of epithelial migration. Furthermore, it was very interesting to see the genes negatively correlated with PRDX6 were enriched in lymphocyte activation.

### Correlations between dy-regulated PRDXs and immune infiltrations in COAD

**Figure 5:**
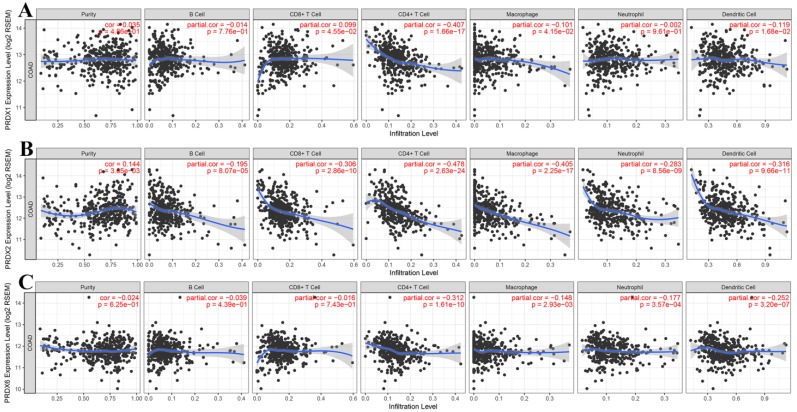
Correlation analyses between dy-regulated PRDXs and immune infiltrations. (A)-(C) represented purity-corrected correlations between dy-regulated PRDXs (PRDX1, PRDX2, and PRDX6) and B cell infiltration, CD8+ T cell infiltration, CD4+ T cell infiltration, macrophage infiltration, neutrophil infiltration, and dendritic cell infiltration, respectively. Partial Spearman's correlation analysis was used and *p* < 0.01 was considered to be significant.

**Figure 6:**
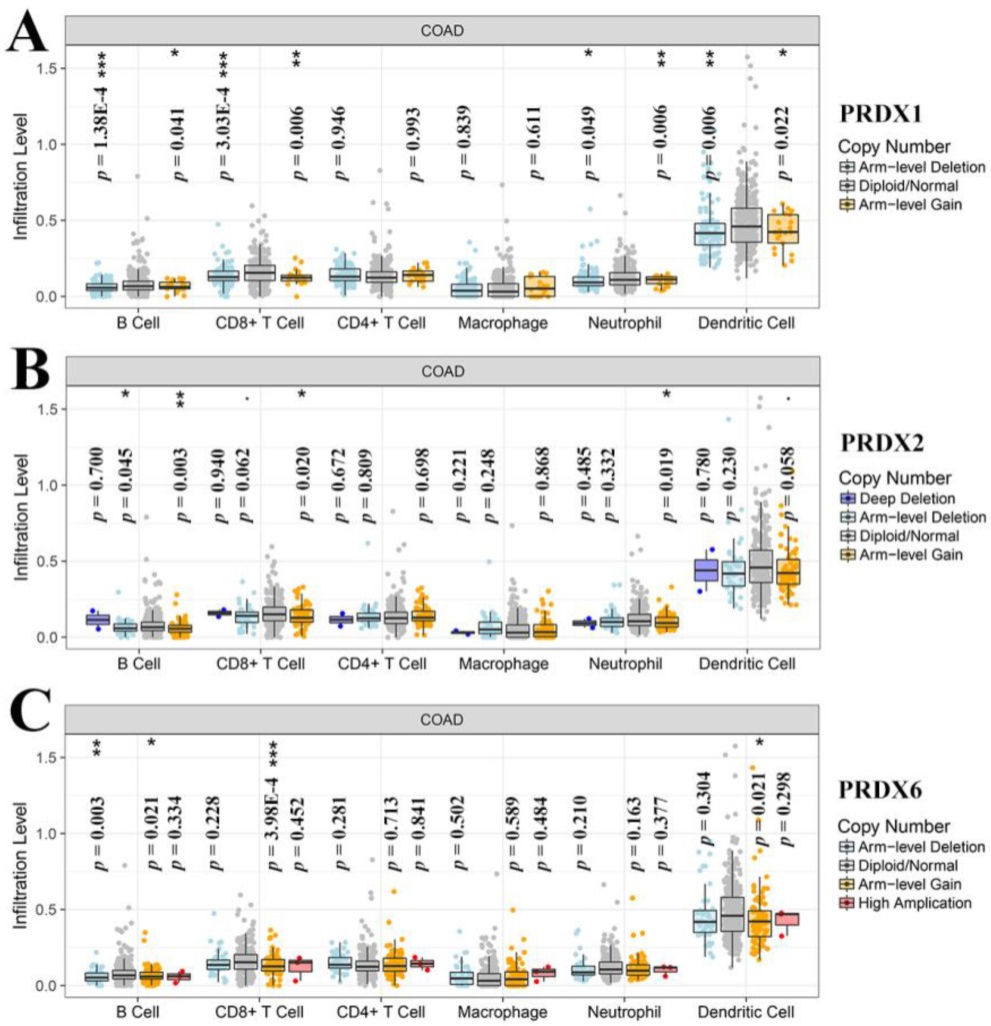
Tumor infiltration level comparisons among COAD tumors with different SCNAs of dy-regulated PRDXs. (A)-(C) represented immune infiltration comparisons among COAD tumors with SCNAs for PRDX1, PRDX2, and PRDX6, respectively. The infiltration level for each SCNA category was compared with the normal using two-sided Wilcoxon rank sum test and *p* < 0.05 was considered to be significant. SCNAs, somatic copy number alterations.

Through online analysis with TIMER, the correlations (partial correlation, adjusted by tumor purity) between the dy-regulated PRDXs (PRDX1, PRDX2, and PRDX6) and the six immune cell infiltration levels were shown and each PRDX was indicated to be negatively correlated with at least one of the immune infiltrations (Figure 5). Noticeably, all the three were significantly negatively correlated with CD4^+^ T cell infiltration (PRDX1: *r* = -0.407, *p* = 1.66E-17; PRDX2: *r* = -0.478, *p* = 2.63E-24; PRDX6: *r* = -0.312, *p* = 1.61E-10). PRDX2 and PRDX6 were also negatively correlated with all and most of the other kinds of immune cell infiltrations, respectively. We also compared the tumor infiltration levels among COADs with different SCNAs of the three PRDXs. As shown in Figure 6, there were differences of B cell and CD8+ T cell infiltration level among COAD tumors with different SCNAs of all the three PRDXs while no significance of CD4^+^ T cell and macrophage infiltration level was shown among the SCNAs of any of them. Interestingly, the immune infiltration levels (with significant difference) were all lower in other SCNAs than that in the normal category, indicating the negative effects of the SCNAs (of the three PRDXs) on the immune response in COAD.

### Prognostic effects of dy-regulated PRDXs and immune infiltration cells in COAD

**Figure 7:**
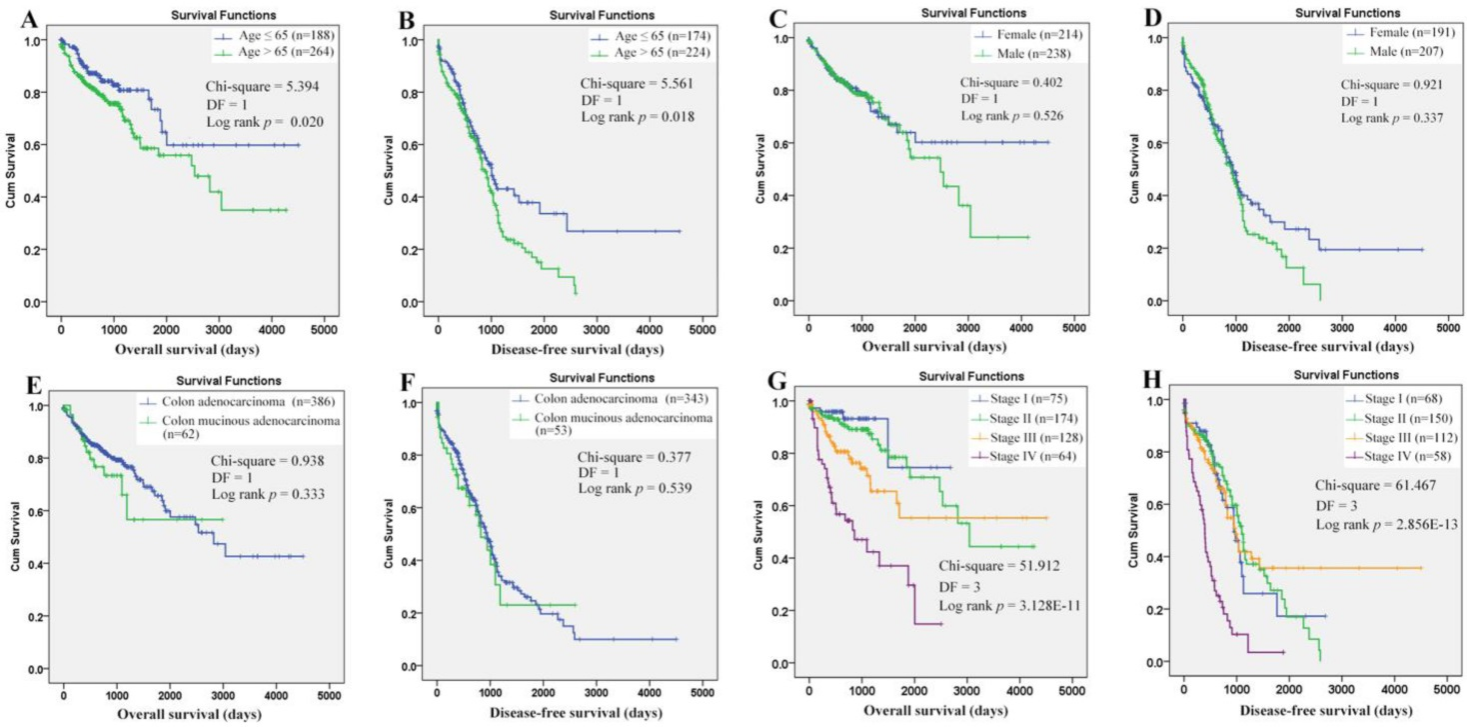
Evaluation of the prognostic roles of the clinical characteristics in overall and recurrence-free survival of COAD patients. (A)-(B), COAD patients with age ≤65 years demonstrated a longer overall and recurrence-free survival than those with age > 65 years; (C)-(D), no significant difference of overall and recurrence-free survival was shown between female and male patients; (E)-(F), no significant prognostic effects of historical type was shown in overall and recurrence-free survival of COAD patients; (G)-(H), there was a significant difference of overall and recurrence-free survival among COAD patients of different TNM stages. Kaplan-Meier survival analysis was used and *p* < 0.05 was considered statistically significant.

Through Kaplan-Meier analysis (Figure 7), a longer OS (*p*=0.020) and RFS (*p*=0.018) was shown in the COAD patients with age (at diagnosis) ≤ 65 years than those with age > 65 years, while no prognostic effects of gender and historical type were shown. The prognostic roles of TNM stage were reported in many COAD studies. Here, its prognostic roles were confirmed (Figure 7G-7H). With TNM stage as strata variable, interestingly, the prognostic effects of age also existed: age>65 years (contrast indicator: age≤65 years) was also shown to be an unfavorable prognostic factor with hazard ratio (HR) 2.034 (95%CI: 1.287-3.214, *p* = 0.002) for OS and HR 1.431 (95%CI: 1.064-1.925, *p*=0.018) for RFS. Considering the survival differences between two age groups (independent of TNM stage), we speculated that there might be some differences in the prognostic effects of the same factors between the younger and older patients. Then, survival analyses were applied to the two groups individually.

**Figure 8:**
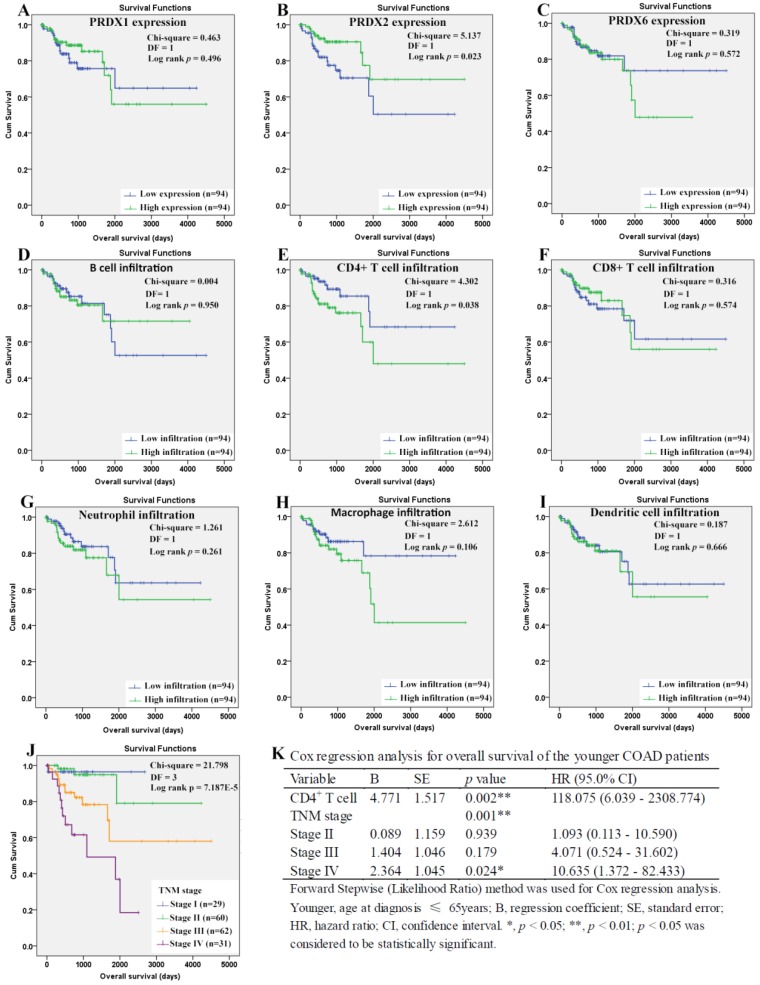
Overall survival analysis of the younger COAD patients (age ≤ 65 years). (A)-(I), overall survival comparisons between the younger COAD patients with low and high expression/infiltration of PRDX1, PRDX2, PRDX6, B cell, CD4+ T cell, CD8+ T cell, neutrophil, macrophage, and dendritic dell, respectively. Kaplan-Meier analysis was used for the comparisons. The median expression (for the four PRDXs) or median infiltration level (for the six kinds of immune cells) was set as the threshold. (J), overall survival comparisons of the younger patients of different TNM stages through Kaplan-Meier survival analysis; (K), multi-variable Cox regression analysis (Forward, likelihood ratio) for overall survival of the younger patients, with age, TNM stage, PRDX2, and CD4+ T cell infiltration as covariates. For all the analyses, *p* < 0.05 was considered to be statistically significant.

**Figure 9:**
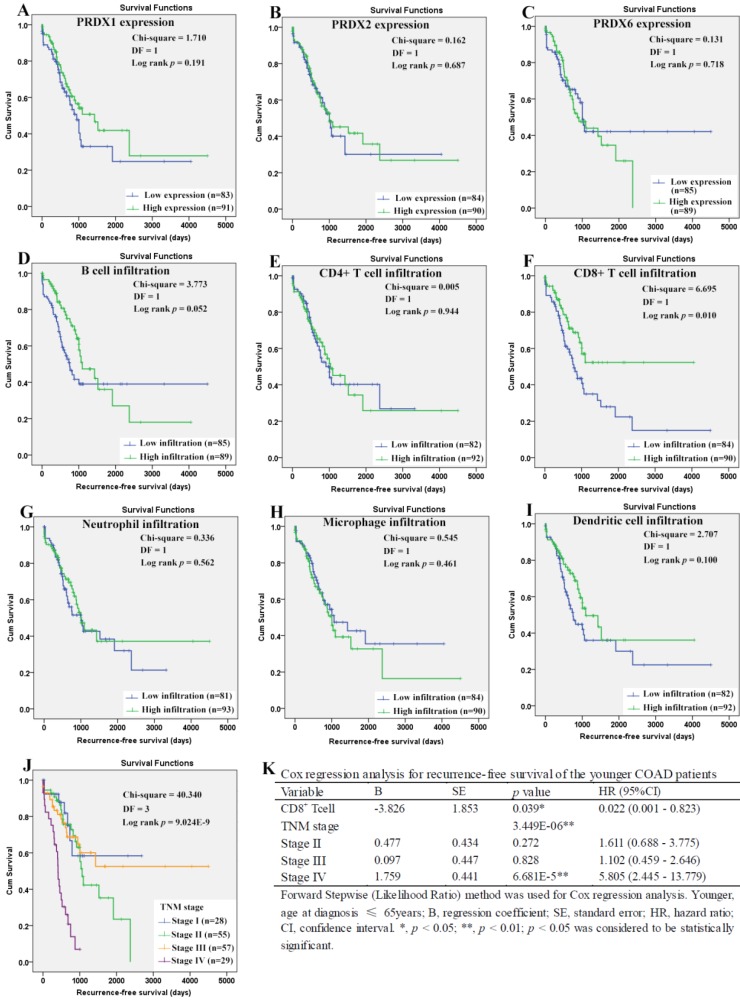
Recurrence-free survival analysis of the younger COAD patients (age ≤ 65 years). (A)-(I), recurrence-free survival comparisons between the younger patients with low and high expression/infiltration of PRDX1, PRDX2, PRDX6, B cell, CD4+ T cell, CD8+ T cell, neutrophil, macrophage, and dendritic dell, respectively. Kaplan-Meier analysis was used for the comparisons. The median expression (for the PRDXs) or median infiltration level (for the immune cells) was set as the threshold. (J), recurrence-free survival comparisons of the younger patients of different TNM stages through Kaplan-Meier survival analysis; (K), multi-variable Cox regression analysis (Forward, likelihood ratio) for recurrence-free survival of the younger patients, with age, TNM stage, and CD8+ T cell infiltration as covariates. For all the analyses, *p* < 0.05 was considered to be significant.

For the younger patients, through Kaplan-Meier analysis, surprisingly, PRDX2 (Figure 8B) which was up-regulated in COAD, was shown to have favorable effects on OS of the younger patients (*p*=0.023). As a shorter OS (*p*=0.038) was seen in the patients with higher infiltration of CD4^+^ T cell (Fig. 8E) comparing with those with lower infiltration, the unfavorable prognostic roles of CD4^+^ T cell was shown. However, none of other two PRDXs (Figures 8A and 8C) and immune cell infiltrations (Figures 8D, 8F-8I) was shown to have significant prognostic functions (*p*>0.05). For TNM stage, its unfavorable effects (*p* = 7.187E-5) on OS of the younger patients were obvious (Figure 8J). With age, TNM stage, PRDX2, and CD4^+^ T cell infiltration as covariates (Figure 8K), through Cox regression analysis, CD4^+^ T cell and TNM stage (stage IV) were indicated to be independent unfavorable prognostic factors for the OS of the younger patients. When it came to RFS of the younger patients (Figure 9), among the three dy-regulated PRDXs and the six immune cells, only CD8^+^ T cell (figure 9F) was found to have favorable effects through Kaplan-Meier survival analysis, with its infiltration level higher, the recurrence-free time longer (*p*=0.010). Similar to its prognostic roles in OS, TNM stage was also shown to be significant in RFS predication for the younger patients (Figure 9J). Through multivariable Cox regression (Figure 9K, with age, TNM stage, and CD8+ T cell infiltration as covariates), CD8+ T cell infiltration (favorable) and TNM stage (stage IV, unfavorable) were shown to be independent RFS predicators for the younger patients. However, age was shown to have no significant effects on the RFS of the younger patients, similar to its roles in the OS.

**Figure 10:**
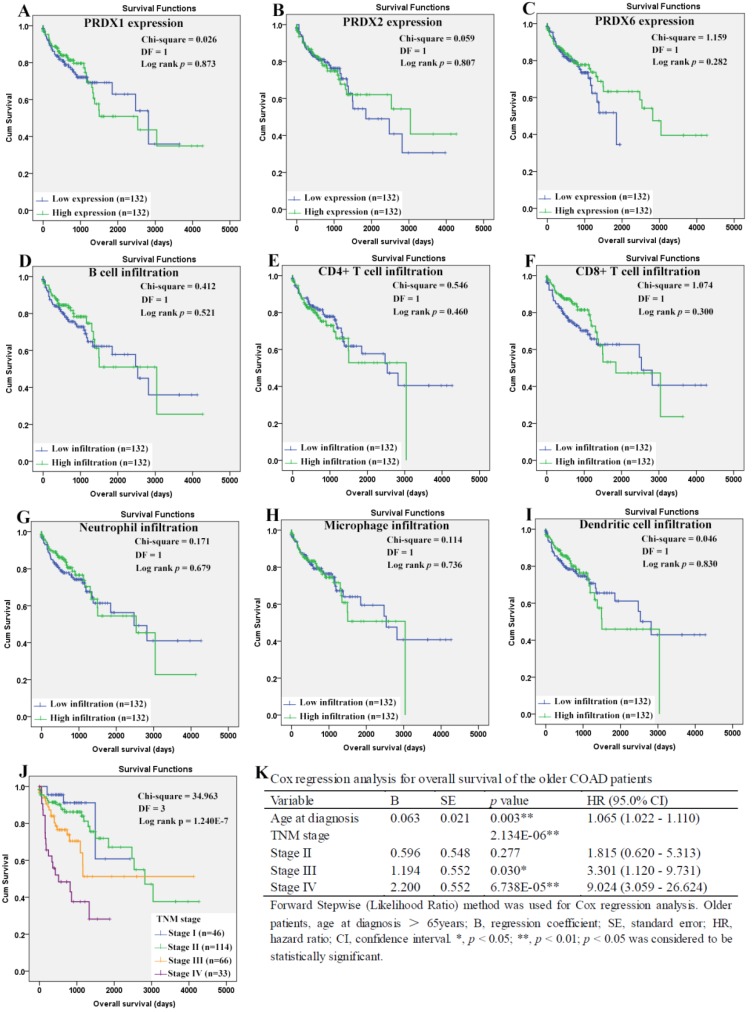
Overall survival analysis of the older COAD patients (age > 65years). (A)-(I), overall survival comparisons between the older patients with low and high expression/infiltration of PRDX1, PRDX2, PRDX6, B cell, CD4+ T cell, CD8+ T cell, neutrophil, macrophage, and dendritic dell, respectively. Kaplan-Meier analysis was used for the comparisons. The median expression (for the PRDXs) or median infiltration level (for the immune cells) was set as the threshold. (J), overall survival comparisons of the older patients of different TNM stages through Kaplan-Meier survival analysis; (K), Cox regression analysis (Forward, likelihood ratio) for overall survival of the older patients, with age and TNM stage as covariates. For all the analyses, *p* < 0.05 was considered significant.

**Figure 11:**
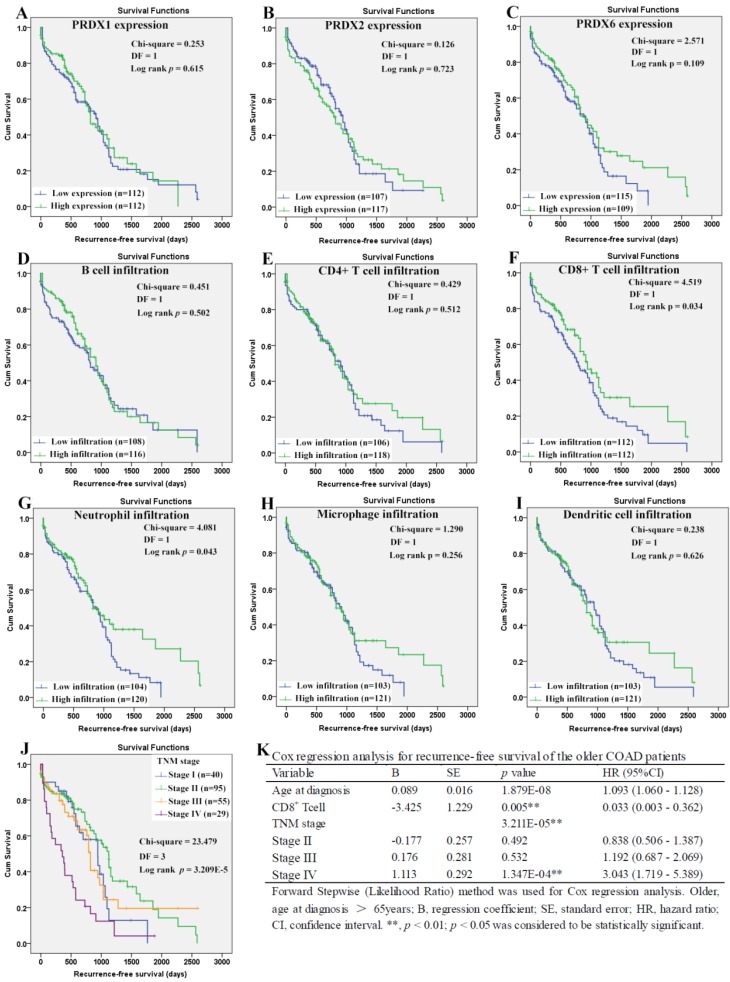
Recurrence-free survival analysis of the older COAD patients (age > 65 years). (A)-(I) represented recurrence-free survival comparisons between the older patients with low and high expression/infiltration of PRDX1, PRDX2, PRDX6, B cell, CD4+ T cell, CD8+ T cell, neutrophil, macrophage, and dendritic dell, respectively. Kaplan-Meier analysis was used for the comparisons. The median expression (for the PRDXs) or median infiltration level (for the immune cells) was set as the threshold. (J), recurrence-free survival comparisons among the older patients of different TNM stages through Kaplan-Meier survival analysis; (K), Cox regression analysis (Forward, likelihood ratio) for recurrence-free survival of the older patients, with age, TNM stage, CD8+ T cell infiltration, and neutrophil infiltration as covariates. For all the analyses, *p* < 0.05 was considered to be statistically significant.

When it came to the older patients, none of the three dy-regulated PRDXs or the six kinds of immune cells was shown to be related to their OS through Kaplan-Meier survival analysis (Figure 10A-10I). With age (continuous variable) and TNM stage (categorical variable) as covariates, the Cox regression model was constructed (figure 10L) and it was shown that age (*p*=0.003), stage III (*p*=0.030), and stage IV (*p*= 6.738E-5) were shown to be independent unfavorable prognostic factors for OS of the older patients with HRs 1.065 (95%CI: 1.022-1.110), 3.301 (95%CI: 1.120- 9.731), and 9.024 (95%CI: 3.059-26.624), respectively. For the RFS of the older patients (Figure 11), comparing with the patients with their low infiltrations, a favorable outcome for the patients with high CD8^+^ T cell infiltration (*p*=0.034) as well as high neutrophil infiltration (*p*=0.043) was shown. While no significant prognostic effects of the PRDXs and the other immune cells was found. Then, CD8^+^ T cell infiltration and neutrophil infiltration, as continuous variables, were used as covariates as well as age (continuous variable) and TNM stage (categorical variable) to construct the Cox regression model for RFS of the older patients. As it was shown in figure 11K, CD8+ T cell infiltration, age, and stage IV were all shown to be independent prognostic factors with HRs 0.033 (95%CI: 0.003-0.362), 1.093 (95%CI: 1.060- 1.128) and 3.043 (95%CI: 1.719-5.389), respectively.

**Figure 12:**
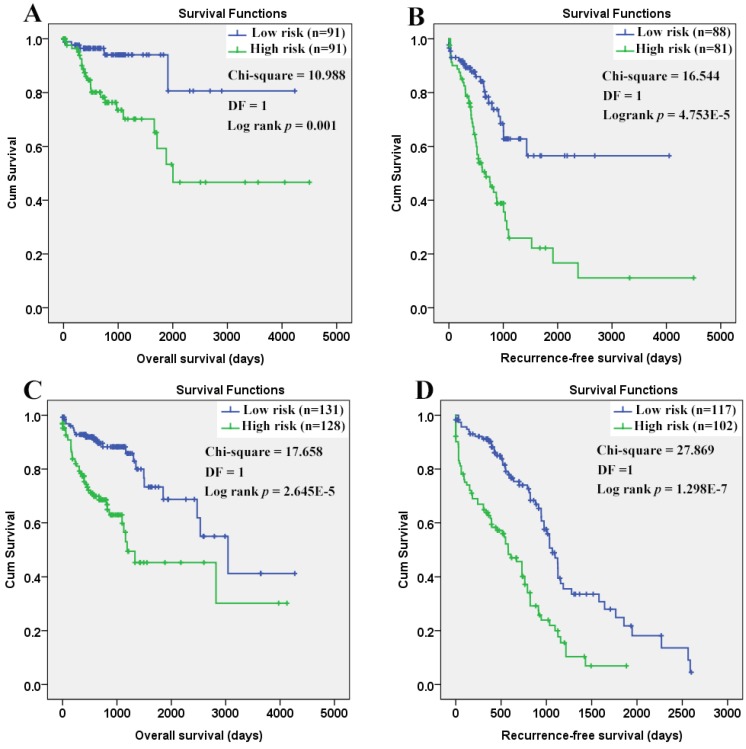
Survival comparisons between the COAD patients with low and high risk scores. (A) Low risk score in the younger patients indicated longer overall survival; (B) Low risk score in the younger patients indicated longer recurrence- free survival; (C) Low risk score in the older patients indicated longer overall survival; (D) Low risk score in the older patients indicated longer recurrence-free survival. The Cox regression models were used to evaluate the risk scores in different age groups individually. Kaplan-Meier survival analyses were applied and with the median risk scores, the patients in each group were divided into low risk group and high risk group. *P* < 0.05 was considered statistically significant.

As shown in figure 12, the efficiency of the Cox models was obvious. Both in the younger and older patients, a longer OS and RFS were shown in the patients with lower risk scores than those with higher.

## Discussion

The involvement of PRDXs in the initiation and progression of human cancer was reported in many studies 13, 15, 61 and some of them were reported to be differentially expressed in colorectal cancers. However, in most of the studies, the PRDXs were studied separately and only the mRNA level or protein level were investigated. Considering the common characteristics of the PRDXs, systemic investigation of them would provide new clues for their roles in physical and pathological processes. Although PRDXs were reported to play important roles in inflammatory disease, the correlations between PRDXs and immune filtrations in COAD and their prognostic effects were unclear. In this study, upon comparisons between tumor and normal samples from COAD patients, we found three of the six PRDXs were dy-regulated in COAD at both mRNA and protein level. The up-regulation of PRDX2, down-regulation of PRDX6, and the nuclear absence of PRDX1 in COAD tumor cells were indicated. Dy-regulations of the PRDX1 and PRDX6 were also confirmed in the stem-like colonospheres from colon cancer cells. Through function enrichments of their correlated genes, the potential roles of the three genes were predicated. For the first time, the negative correlations between the three dy-regulated PRDXs and the immune infiltrations in COAD were found and their prognostic effects were investigated. The Cox regression models for OS and RFS in different age groups were constructed and they could discriminate the low- and high-risk patients well.

PRDX1, firstly known for its antioxidant activities, also named NKEF-A or PAG, could enhance the cytotoxicity of natural killer cells 62 and function as tumor suppressor 63, 64. However, its tumor- promoting effects were also shown in many tumors 15,24,34,65, indicating its dual roles in malignances. In colorectal cancer, PRDX1 was also reported to be overexpressed in the tumor tissues at protein level in previous studies 32, 34 with predominantly cytoplasmic localization 34. Similarly, in breast cancer 66, low nuclear but high cytoplasmic PRDX1 expression was also demonstrated. In this study, although PRDX1 was shown to be up-regulated in COAD at mRNA level, its increase was not confirmed at protein level. However, the nuclear absence of PRDX1 in COAD tumor cells was consistent. Considering the protective effects of PRDX1 against oxidative damage at telomeres 67, 68, we speculated that the loss of PRDX1 in nucleus might lead to telomere crisis and genome instability which could promote tumor progression 69-71.

As an antioxidant enzyme, the protective roles of PRDX2 against oxidative stress-induced cell death were shown in many studies 72-74. In colorectal cancer, as the most abundant isoform of the PRDXs in the tumor tissues 75, PRDX2 was reported to be a tumor-promoter in colorectal cancers with APC mutation 76. In a previous study 36, PRDX2 was shown to be elevated in colorectal cancer tissues compared to the normal controls. Here, the up-regulation of PRDX2 in COAD was confirmed both at mRNA and protein levels. Considering the important roles of PRDX2 in resistance of human colorectal cancer cells to chemotherapy and radiotherapy 77, 78, it might be a new target for COAD therapy.

With regard to PRDX6, its over-expression was associated with carcinogen-induced tumor incidence 79 and tumor-progression 80 in lung cancer. However, in hepatocellular carcinoma, PRDX6 was lower expressed in the tumor than the non-tumor tissues and its decrease was associated with poor prognosis 25, indicating its anti-tumor function in liver cancer. In COAD, although its tumor-promoting effects were reported in one study^81^, there were more evidence to support its anti-tumor functions. In our previous study 82, we have evaluated the serum level of PRDX6 and it was shown to be higher in lung cancer while lower in colon cancer than their healthy controls. Here, down-regulation of PRDX6 in COAD was confirmed both at mRNA and protein level and its under-expression in the colonospheres form primary colon cancer cells were shown. Since it was reported that PRDX6 was essential for anti-tumor effects of baicalein and the up-regulation of PRDX6 was associated with growth inhibition of colorectal cancer cells 83, we speculated that PRDX6 might be a tumor-suppressor during COAD development and it might be a potential target for chemotherapy.

Gene co-expression network was demonstrated to be useful for uncovering potential functions of specific genes 84-86. The important roles of PRDX1 in autophagy activation were shown in many studies 87, 88. Here, through the enrichments of its positively correlated genes, PRDX1 was shown to be associated with autophagy and the reliability of the method was indicated. The importance of mitochondrial homeostasis for normal RNA metabolism were reported 89-91 and the protective effects of PRDX1 and PRDX6 on mitochondria were demonstrated 92, 93 in many studies. However, the roles of abnormal RNA metabolism and mitochondrial-related changes in the development of COAD were unclear. In this study, RNA metabolism and mitochondrial related processes were highlighted to be common processes that all the three dy-regulated PRDXs might be associated, providing new clues for the study of COAD pathogenesis. In liver cancer 26, 94, lung cancer 95, and breast cancer 18, the associations between PRDXs and Ras signaling pathway have been demonstrated. Here, the Ras protein signal transduction was also enriched by the negatively correlated genes of PRDX1 and PRDX2. Considering the associations between Ras activation and the up-regulation of intracellular reactive oxygen species (ROS) 96 and the antioxidant characters of the PRDXs, it was speculated that they might play different roles in ROS regulation and their correlations during COAD progression needed further investigation. The roles of PRDXs in inflammation were reported in previous studies 41, 97. Here, the genes negatively correlated PRDX6 were shown to be associated with lymphocyte activation and all the three dy-regulated PRDXs were also shown to be negatively correlated with CD4^+^ T cell infiltration in COAD, indicating their potential roles in the regulation of immune response during COAD progression.

The importance of immune response was shown in many tumors 98-101. In lung cancer, CD4^+^ T cells were demonstrated to be associated with its progression and metastasis 102. In breast cancer 103 and bladder cancer 104, tumor-infiltrating naive CD4^+^ T cells were correlated with poor survival. In COAD, depletion of CD4^+^CD25^+^ regulatory T cells was reported to be able to enhance interleukin-2-induced antitumor immunity in a mouse model^105^ and lower CD4^+^/CD8^+^ ratio of the tumor infiltrating lymphocytes was demonstrated to be associated with better survival of colorectal cancer patients^106^, indicating the unfavorable effects of CD4^+^ T cell. However, here, upon the Cox regression models for COAD patients, the similar roles of CD4^+^ T cell infiltration in COAD were found only in the younger patients while no significance was shown in the older patients, indicating the different prognostic effects of the same factor in different age groups. The unfavorable prognostic effects of age were reported in many tumors including thyroid cancer 107, salivary gland carcinoma 108, prostate cancer and colon cancer 109. Here, age was also shown to be prognostic in COAD patients. However, when grouping the patients according to their age at diagnosis, its significant unfavorable effects on OS and RFS were only shown in the older patients. We speculated that there might be more negative effects of aging on the older patients and age should be considered in COAD treatment. In contrast to CD4^+^ T cell and age at diagnosis, similar prognostic effects of TNM stage and CD8^+^ T cell were shown in COAD patients of different age groups. TNM stage, especially stage IV, was independent prognostic factor for OS and RFS of both younger and older patients, indicating the importance of early diagnosis. As for CD8^+^ T cell, its enhanced activation was associated with improved survival in many tumors including gastric cancer 110, hepatocellular carcinoma 111, breast cancer 112, and head and neck cancers 113. Here, the protective effects of CD8^+^ T cell in preventing tumor recurrence were shown in both the younger and the older COAD patients, consistent with its favorable prognostic effects in colorectal cancer in previous studies 114-116, indicating its anti-tumor property during COAD progression and this would provide a basis for its application in COAD immunotherapy.

## Conclusion

In summary, the dy-regulation of PRDX1, PRDX2, and PRDX6 were identified and confirmed both at mRNA and protein level, ensured the reliability of the results. The potential roles of the PRDXs and their correlations with tumor immune infiltrations might provide new clues for the study of COAD occurrence and progression. Although no significant independent prognostic effects of the three PRDXs were shown, the significant differences in their expressions (PRDX2 and PRDX6) or locations (PRDX1 and PRDX6) indicated their potential as new markers for COAD diagnosis. Considering the negative correlations between the dy-regulated PRDXs and the immune infiltrations, the potential roles of PRDXs in immunoregulation were indicated. For the first time, we considered the effects of age and analyzed the survival of the younger and older patients separately. The unfavorable prognostic effects of CD4^+^ T cell infiltration on OS in the younger patients indicated its potential roles as therapeutic target in the COAD treatments. The protective roles of CD8^+^ T cells might provide new direction for preventing tumor replase. However, we also have limitations in our study, although the expressional differences of the PRDXs were shown, their specific roles in COAD development were unclear. Whether their expressional changes are the cause or the outcome of COAD occurrence needs further study. To further evaluate the diagnostic power of the dy- regulated PRDXs and their potential roles in COAD immune-regulation, large scale investigation is need. The values of CD4^+^ T cell and CD8^+^ T cell in immunotherapy of COAD also need further exploration.

## Figures and Tables

**Table 1:** Clinicopathological features of the 452 COAD patients from TCGA database

Character	Case, n ( %)
**Age at diagnosis (yr)**	
≤65	188 (41.6%)
>65	264 (58.4%)
**Gender**	
Male	238 (52.7%)
Female	214 (47.3%)
**TNM stage**	
Stage Ⅰ	75 (16.6%)
Stage Ⅱ	174 (38.5%)
Stage Ⅲ	128 (28.3%)
Stage Ⅳ	64 (14.2%)
NA	11 (2.4%)
**Historical type**	
Colon adenocarcinoma	386 (85.4%)
Colon mucinous adenocarcinoma	62 (13.7)
NA or discrepancy	4 (0.9%)
**Overall survival status**	
Alive	354 (78.3%)
Dead	98 (21.7%)
**Recurrence status**	
Yes	204 (45.1%)
No	197 (43.6%)
Not available	51 (11.3%)

NA, not available.

**Table 2:** Expressional differences of PRDXs in COAD datasets from Oncomine database

Dataset	Comparison	PRDX1	PRDX2	PRDX4	PRDX6
Notterman Colon (n=36)	Colon adenocarcinoma vs. normal	FC = 1.657 *p* = 3.75E-5^**^	FC = 2.483 *p*= 0.004^**^	FC = 2.745 *p* = 6.13E-7^**^	FC = -1.748 *p* = 2.36E-4^**^
Alon Colon (n=62)	Colon adenocarcinoma vs. normal	FC = 1.421 *p* = 0.004^**^	FC = 2.483 *p* = 0.004^**^	NA	FC = -1.529 *p* = 5.30E-4^**^
Ki Colon (n=123)	Colon adenocarcinoma vs. normal	FC = 1.387 *p* = 3.05E-6^**^	FC = 1.102 *p* = 0.154	FC = 1.294 *p* = 8.23E-4^**^	FC = -2.231 *p* = 7.31E-15^**^
Kaiser Colon (n=105)	Colon adenocarcinoma vs. normal	FC = 1.326 *p* = 0.001^**^	FC = 1.322 *p* = 0.002^**^	FC = 2.033 *p* = 8.13E-5^**^	FC = -1.830 *p* = 0.005^**^

NA, not available; FC, fold change. *, *p* < 0.05; **, *p* < 0.01; *p* < 0.05 was considered to be statistically significant.

**Table 3:** Expressional differences of dy-regulated PRDXs between colonospheres from HT29 cell line and their parental cells.

Probe ID	Gene symbol	Adj. *p*	*p* value	t	B	logFC
208680_at	PRDX1	0.005**	0.0004	5.612	0.102	0.838
215067_x_at	PRDX2	0.130	0.035	2.490	-4.630	0.380
39729_at	PRDX2	0.287	0.115	-1.753	-5.764	-0.264
211658_at	PRDX2	0.770	0.591	-0.559	-7.051	-0.101
201006_at	PRDX2	0.855	0.724	-0.365	-7.148	-0.069
200845_s_at	PRDX6	0.006**	0.0005	-5.319	-0.291	-0.813
200844_s_at	PRDX6	0.235	0.084	-1.948	-5.477	-0.297

Adj.*p*, adjusted *p* value; logFC, log_2_ fold change. Benjamini and Hochberg (the false discovery rate) was applied to adjust the *p* values. **, adj.*p*<0.01, adj.*p*<0.05 was considered significant.

## Abbreviations

PRDXs: peroxiredoxins
COAD: colon adenocarcinoma
TPM: transcripts per million
HPA: Human Protein Atlas
TIMER: Tumor Immune Estimation Resource
OS: overall survival
RFS: recurrence-free survival
iPLA2: Ca2+-independent phospholipase A2
IHC: immunohistochemical
